# Myocardial ischemia-reperfusion injury upregulates nucleostemin expression via HIF-1α and c-Jun pathways and alleviates apoptosis by promoting autophagy

**DOI:** 10.1038/s41420-024-02221-x

**Published:** 2024-10-30

**Authors:** Xiao Han, Zhicheng Jiang, Yufeng Hou, Xiaorong Zhou, Baoying Hu

**Affiliations:** 1 Department of Cardiothoracic Surgery, Affiliated Hospital of Nantong University & Department of Immunology, Medical School of Nantong University, Nantong, 226001 People’s Republic of China; 2 Department of Immunology, Medical School of Nantong University, Nantong, 226001 People’s Republic of China

**Keywords:** Myocardial infarction, Chaperone-mediated autophagy

## Abstract

Myocardial ischemia-reperfusion (I/R) injury, often arising from interventional therapy for acute myocardial infarction, leads to irreversible myocardial cell death. While previous studies indicate that nucleostemin (NS) is induced by myocardial I/R injury and mitigates myocardial cell apoptosis, the underlying mechanisms are poorly understood. Here, our study reveals that NS upregulation is critical for preventing cardiomyocyte death following myocardial I/R injury. Elevated NS protein levels were observed in myocardial I/R injury mouse and rat models, as well as Hypoxia/reoxygenation (H/R) cardiac cell lines (H9C2 cells). We identified binding sites for c-Jun and HIF-1α in the NS promoter region. Inhibition of JNK and HIF-1α led to a significant decrease in NS transcription and protein expression. Furthermore, inhibition of autophagy and NS expression promoted myocardial cell apoptosis in H/R. Notably, the cell model showed reduced LC3I transformation to LC3II, downregulated Beclin1, upregulated p62, and altered expression of autophagy-related proteins upon NS interference in H/R cells. These findings suggest that NS expression, driven by c-Jun and HIF-1α pathways, facilitates autophagy, providing protection against both myocardial I/R injury and H/R-induced cardiomyocyte apoptosis.

## Introduction

Cardiovascular disease (CVD) stands as the leading cause of global morbidity (523 million), mortality (18.6 million), and disability (34.4 million), placing a substantial economic burden on healthcare systems worldwide. Notably, China bears the highest number of CVD-related deaths [1]. Myocardial ischemia/reperfusion (I/R) injury represents a critical concern in cardiovascular medicine, commonly arising as a consequence of interventional coronary procedures or acute myocardial infarction. This phenomenon occurs when the blood supply to the heart muscle is temporarily restricted and then restored, triggering a cascade of events, including local inflammation, a hypoxic environment, myocardial dysfunction, and structural damage, culminating in secondary injury that can lead to additional damage [2]. Myocardial I/R injury is a multifaceted process involving complex interactions among various cellular and molecular pathways, including the JNK/c-Jun pathway [3–7], the Hypoxia-inducible factor-1 (HIF-1) signal pathway [8–10], and the autophagy signal pathway [11–14]. Despite intensive investigations, the roles of these pathways during myocardial I/R injury remain controversial. Thus, elucidating the molecular mechanisms underlying myocardial I/R injury is pivotal for developing targeted interventions and therapies to mitigate its impact on cardiac function and improve patient outcomes [15, 16].

Nucleostemin (NS) is a protein mainly located in the nucleolus and also known as guanine-nucleotide-binding protein-like 3 (GNL3). It contains a unique MMR1-HSR1 structural domain composed of five GTP binding motifs arranged in a circular order. Due to its structure, NS plays a vital role in preventing DNA replication damage, regulating cell-cycle progression, cell growth, and self-renewal, as well as other biological processes [17–19]. NS was initially discovered to be highly expressed in neuroepithelial stem cells and progenitor cells but was later found to be enriched in other types of stem cells and tumour cells [20–22]. However, its expression declines rapidly in differentiated cells and tissues [23]. Mark Sussman’s lab, for the first time, discovered that NS expression can be induced by a myocardial infarct [24]. Moreover, NS was not only instantaneously upregulated in the rat cardiomyocytes and cardiac progenitor cells exposed to doxorubicin or actinomycin D, but also detected to be transiently highly expressed in the Myocardial I/R injury. Previous studies have reported upregulation of NS expression can protect against cell apoptosis by inhibiting the activation of caspase and p53 [25, 26]. However, whether NS participates in myocardial I/R injury development and alleviates secondary cardiomyocyte damage remains largely elusive.

In this study, we investigated the expression and effects of NS in mouse and cell line models of hypoxia/reoxygenation (H/R). We also explored the pathways that triggers the expression of NS. Moreover, we applied interference of NS to verify the downstream autophagy pathway and its impact on H9C2. These findings suggest that NS could be a promising therapeutic target for reducing secondary cardiomyocyte damage after myocardial I/R injury.

## Results

### Myocardial I/R injury-induced NS expression

**Fig. 1:**
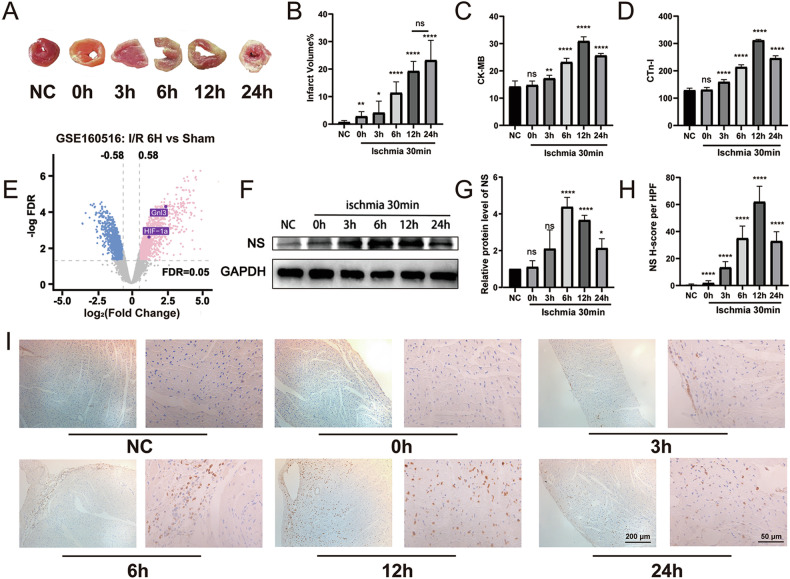
Myocardial I/R injury induces upregulation of NS protein expression. **A**, **B** Representative images (**A**) and quantitative analysis (**B**) of TTC-stained heart sections following 30 min of LAD occlusion and subsequent reperfusion for 0, 3, 6, 12, and 24 h (*n* = 3). **C**, **D** Evaluation of infarction indicators, CK-MB, and CTn-I using ELISA (*n* = 3). **E** Volcano plot analysis of the GSE160516 dataset depicting gene expression differences between ischemia 30 min/reperfusion 6 h samples and sham samples (*n* = 4; FDR < 0.05 and |log2FC| > 0.58). **F**, **G** Western blot and quantitative analysis of NS protein expression at various reperfusion time points after 30 min of ischemia (*n* = 3). **H**, **I** Immunohistochemistry and quantification of the positive rate of NS. *P < 0.05, **P < 0.01, ***P < 0.001, ****P < 0.0001, vs. NC group.

To establish a suitable ischemia-reperfusion (I/R) animal model, TTC staining was employed to assess myocardial ischemia severity. Additionally, we utilized ELISA to gauge cardiac enzyme indicators, including CTn-I and CK-MB, at various time points (0, 3, 6, 12, and 24 h) post 30 min of ischemia followed by reperfusion. The myocardial infarction area showed a significant increase over time after I/R (Fig. 1A, B), and both CTn-I and CK-MB levels followed a similar temporal pattern, peaking at 12 h and diminishing thereafter (Fig. 1C, D). These findings validated the successful construction of the I/R model.

To identify key differential genes between the I/R injury and sham-operation groups, we analyzed three GEO databases (GSE160516, GSE122020, and GSE58486). The consistent outcome across all databases highlighted the significant upregulation of the Gnl3 gene encoding the NS protein in the I/R group (Figs. 1E and S1A). Subsequently, to assess NS expression profile during cardiac I/R injury, we conducted western blot and immunohistochemistry analyses on various tissue groups. Our findings unveiled a swift elevation in NS protein levels, peaking at 12 h after 3 h of reperfusion, and then declining by 24 h compared to the normal control group. This pattern was consistent with the mRNA expression level of NS observed in the GSE160516 RNA-seq dataset (Figs. 1F, G and S1B). Moreover, the positive rate of NS was consistent with the protein level (Fig. 1H, I). Collectively, these findings indicate that the temporal expression of NS may play a crucial role in myocardial I/R injury.

### CoCl_2_-mediated hypoxia and reoxygenation upregulated NS expression in H9C2 cells

**Fig. 2:**
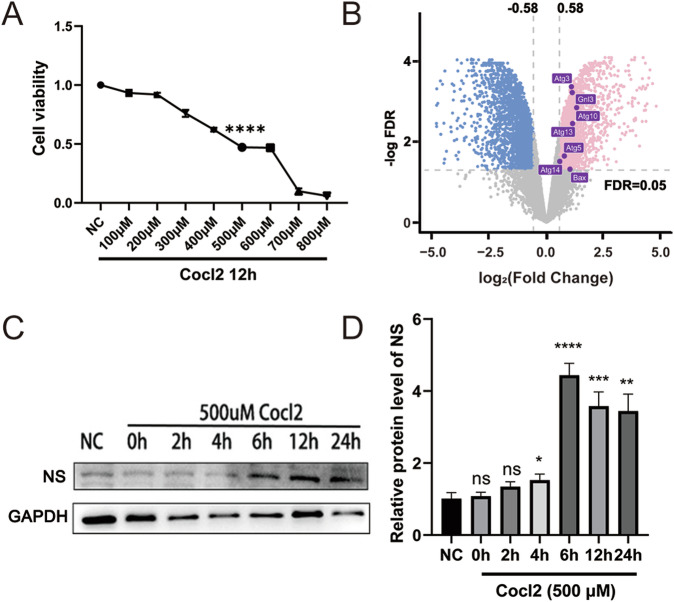
NS is upregulated following CoCl_2_-induced hypoxia and reoxygenation in H9C2 cells. **A** Assessment of CoCl_2_-induced H9C2 cell death at various concentrations using CCK8. **B** Volcano plot analysis of RNA-seq data in H9C2 cells between hypoxia 12 h/reperfusion 6 h and sham samples (*n* = 3, FDR < 0.05 and |log2FC| > 0.58). **C**, **D** Western blot assays depicting the expression of NS after 12 h of hypoxia induced by 500 μM CoCl_2_ and reperfusion for 0, 2, 4, 6, 12, and 24 h (*n* = 3). *P < 0.05, **P < 0.01, ***P < 0.001, ****P < 0.0001, vs. NC group.

The CCK8 results demonstrated that the cell viability of H9C2 was ~50% following 12 h of exposure to a hypoxic and glucose-deficient environment simulated by glucose-free DMEM supplemented with 500 μM CoCl_2_ in vitro (Fig. 2A). RNA-seq analysis of the cell model further substantiated the elevation of NS during H/R (Fig. 2B). NS protein expression, resembling the tissue level, exhibited a significant upregulation, reached its peak at 6 h of reoxygenation, and declined subsequently at 12 h (Fig. 2C, D). In summary, our findings strongly suggest that NS expression might be intricately associated with the process of myocardial cell hypoxia-reperfusion.

### C-Jun and HIF-1α bind directly to the promoter region of NS

**Fig. 3:**
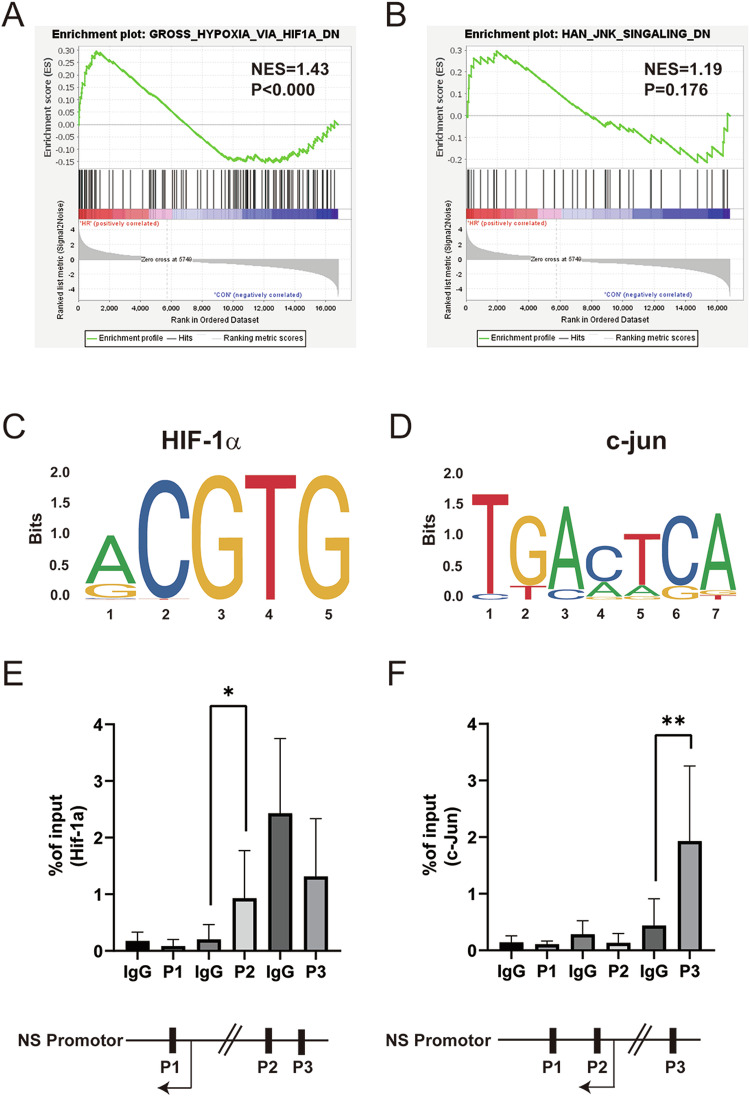
c-Jun and HIF-1α directly bind to the NS promoter regions. **A**, **B** GSEA enrichment analysis of RNA-seq data, HIF1A, and JNK pathway were enriched. **C**, **D** HIF-1α and c-Jun potential binding base sites in the NS promoter region were predicted by the Jaspar tool. **E**, **F** HIF-1α and c-Jun binding to NS promoter regions were tested by CHIP-qPCR. (*n* = 3). *P < 0.05, **P < 0.01, *vs*. IgG group.

Subsequently, we investigated the potential mechanisms underlying NS expression in both I/R and H/R. Further insight was gained by characterizing the transcriptomic profile of the H12h/R 6 h group vs Sham group. GSEA enrichment analysis highlighted the activation of the GROSS_HYPOXIA_HIF1A_DN pathway and the HAN_JNK_SINGALING_DN pathway (Fig. 3A, B). Utilizing the JASPAR tool, we predicted several binding sites of c-Jun and HIF-1α in the NS promoter region (Fig. 3C, D). Consequently, ChIP was employed to precipitate DNA fragments bound to c-Jun and HIF-1α, and the subsequent qPCR results indicated the presence of both c-Jun and HIF-1α binding at the NS promoter region (Fig. 3E, F). These findings strongly suggest that NS might be regulated by the HIF-1α and the JNK/c-Jun pathways.

### The JNK/c-Jun pathway and HIF-1α pathway drive the expression of NS during cardiac H/R injury

**Fig. 4:**
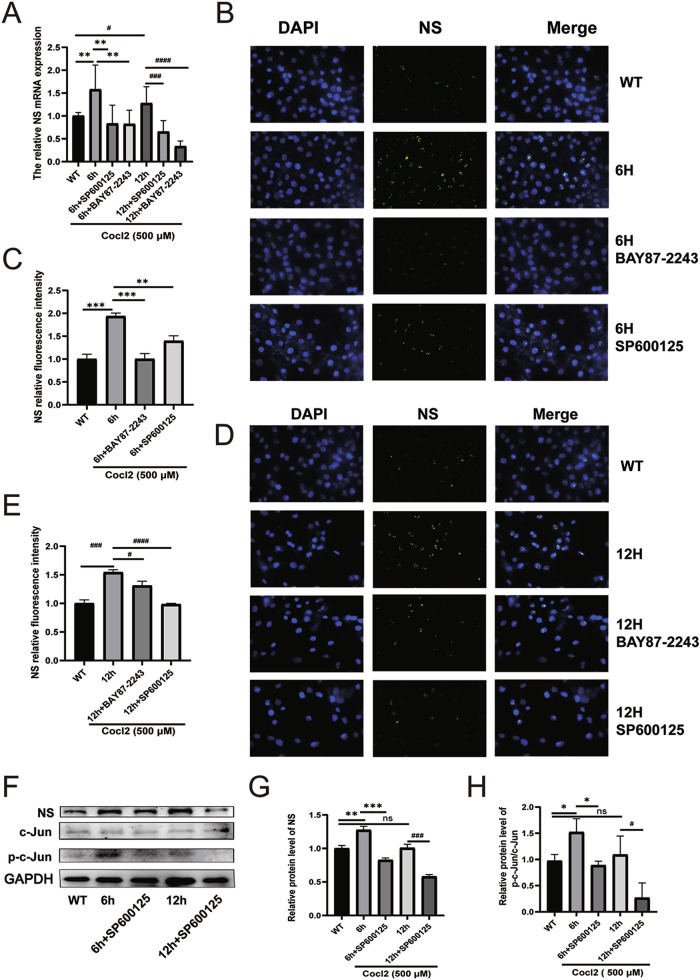
JNK/c-Jun and HIF-1α pathways upregulate the expression of NS in H/R. **A** NS mRNA level was tested following hypoxia/reoxygenation in the absence or presence of JNK and HIF-1α inhibitors. **B**–**E** Immunofluorescence was applied to examine and quantify the expression of NS (green) in the absence or presence of JNK and HIF-1α inhibitors after 12 h of hypoxia and 6 or 12 h of reoxygenation. **F**, **H** western blot assays showed the expression of NS and p-c-Jun protein levels with or without exposure to JNK inhibitor SP600125 during hypoxia/reoxygenation. (*n* = 3). *P < 0.05, **P < 0.01, ***P < 0.001, ****P < 0.0001, vs. 6 h group. ^#^*P* < 0.05, ^###^*P* < 0.001, ^####^*P* < 0.0001, vs. 12 h group.

To investigate the regulatory impact of c-Jun and HIF-1α on NS expression during H/R, inhibitors of JNK (SP600125) and HIF-1α (BAY87-2243) were administered. The qPCR results revealed a downregulation in the mRNA level of NS at 6 and 12 h of reoxygenation following exposure to JNK and HIF-1α inhibitors (Fig. 4A). Consistently, a parallel reduction in the fluorescence intensity of NS was observed (Fig. 4B–E). Notably, c-Jun phosphorylation peaked at 6 h and then declined at 12 h post-reoxygenation. Concurrently, treatment with JNK inhibitor SP600125 downregulated NS expression at both time points, suggesting a causal role of JNK/c-Jun activation in the regulation of NS during H/R injury (Fig. 4F–H).

### NS alleviates apoptosis in H9C2 cells under H/R injury

**Fig. 5:**
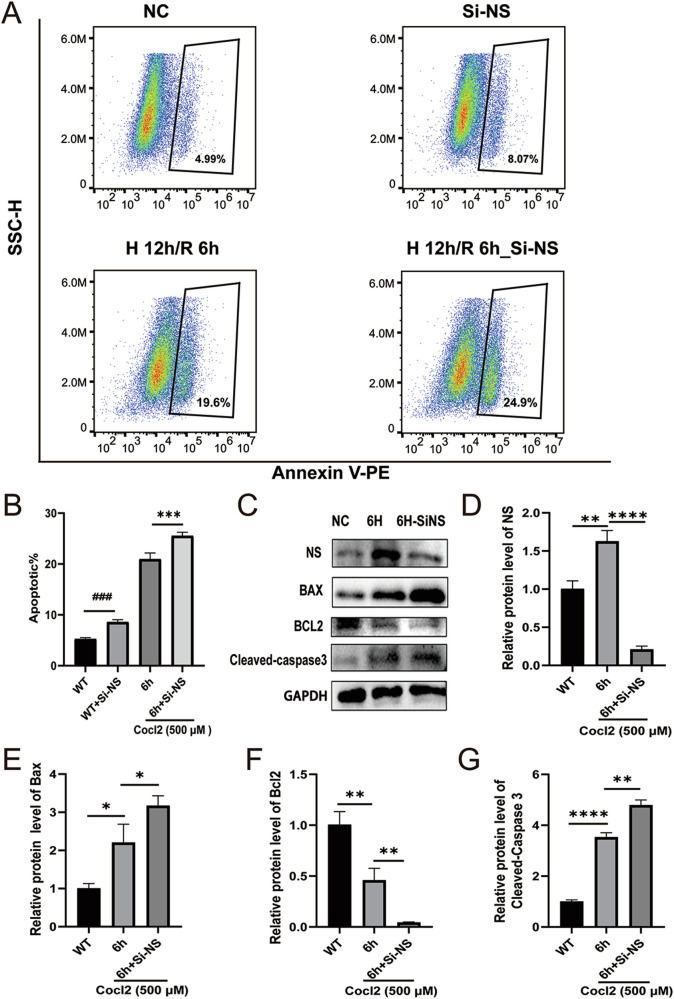
NS alleviates the apoptosis of H9C2 cells under H/R injury. **A**, **B** Flow cytometry analysis of H9C2 cell apoptosis with or without NS interference after 12 h of hypoxia and 6 h of reoxygenation. **C**–**G** Western blot analysis of apoptosis-related proteins, NS, Bax, Bcl2, Cleaved Caspase-3, following NS interference during hypoxia/reoxygenation injury. *P < 0.05, **P < 0.01, ***P < 0.001, ****P < 0.0001, vs. 6 h group. ^###^*P* < 0.001, vs. WT group.

Next, we examined the protective effect of NS expression during cardiac H/R injury. As predicted, flow cytometry analysis showed that interfering with NS deteriorates H/R-induced cell apoptosis (Fig. 5A, B). Compared with cells transduced with non-targeting control siRNA (NC), depletion of NS using an NS siRNA pool led to the upregulation of pro-apoptotic Bax and Cleaved Caspase-3, but downregulated the level of anti-apoptotic Bcl2 at 6 h after reoxygenation, underscoring the importance of NS upregulation in preventing apoptotic cell death during H/R injury (Fig. 5C–G).

### NS facilitates autophagy to mitigate apoptosis in H9C2 cells

**Fig. 6:**
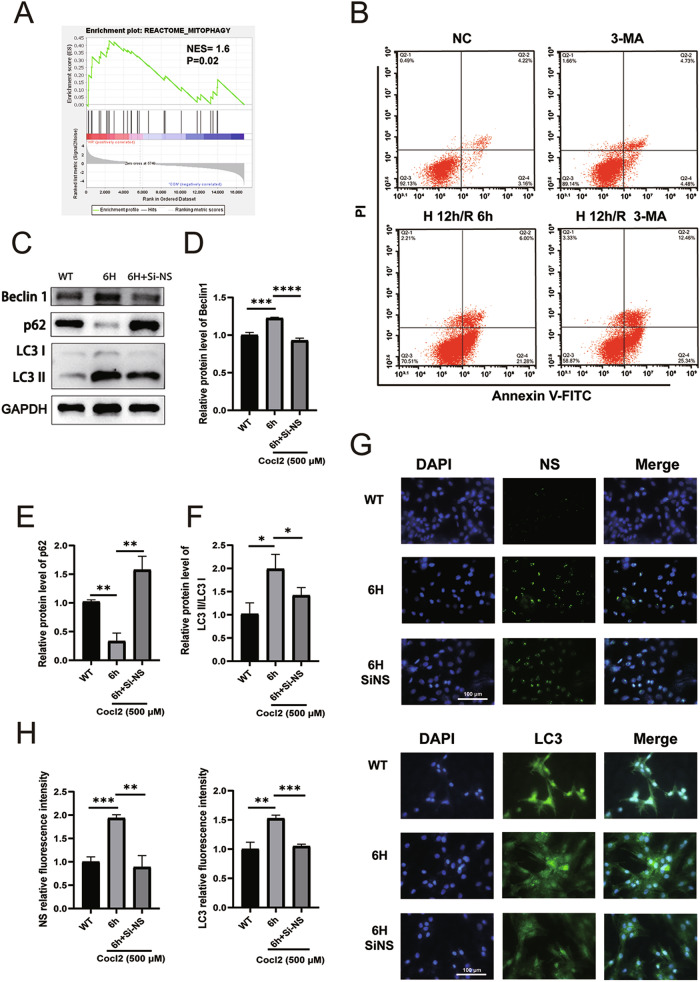
NS protects H9C2 cells from H/R injury by promoting autophagy. **A** Autophagy-related pathway was enriched after H/R injury using GSEA. **B** Representative images of flow cytometry analysis depict the apoptosis rates in the indicated groups. **C**–**F** Autophagy-related proteins, Beclin1, p62, LC3I, and LC3II, were detected by western blot analysis after interfering with NS and H/R. **G**, **H** NS and LC3 were detected by immunofluorescence after interfering with NS and H/R and quantified. *P < 0.05, **P < 0.01, ***P < 0.001, ****P < 0.0001, vs. 6 h group.

To further dissect the molecular mechanism by which NS upregulation protects cardiomyocytes from H/R injury, we interrogated the signaling pathway involved in H/R injury using GSEA analysis, and revealed an enrichment in the REACTOME_MITOPHAGY pathway (Fig. 6A). Given the ongoing debate regarding the involvement of autophagy in H/R injury, we introduced the autophagy inhibitor 3-MA in H9C2 H/R injury model. Notably, inhibition of autophagy using 3-MA significantly deteriorated the apoptotic death of H9C2 cardiomyocytes, compared to the control group, suggesting a protective role of autophagy in H9C2 cardiomyocytes following 12 h of CoCl_2_-induced hypoxia and 6 h of reoxygenation (Fig. 5B).

Furthermore, Western blot analysis confirmed that interference with NS during H/R injury resulted in diminished autophagy, characterized by reduced transformation from LC3I to LC3II, upregulation of p62, and downregulation of Beclin1 (Fig. 5C–F). Additionally, the fluorescence intensity of LC3 was markedly reduced under NS interference (Fig. 5G, H). Taken together, these findings indicate that NS upregulation can alleviate H/R-induced apoptosis by initiating autophagy in H9C2 cells.

## Discussion

In this study, we substantiated a potential mechanism in which JNK/c-Jun and HIF-1α pathways upregulate NS expression, promoting autophagy and mitigating cell apoptosis. This mechanism sheds light on the transient expression’s protective effect of NS in cardiomyocytes undergoing I/R injury.

The irreversible damage caused by myocardial I/R injury may be attributed to the limited regenerative capacity of terminally differentiated myocardial cells, resulting in apoptosis [27]. Previous studies have identified NS as a stem-related protective protein during genotoxic stress, ROS injury, and aging [28–30]. Consistent with previous reports, our results demonstrate an immediate upregulation of NS expression after 6 h of reperfusion, followed by a decline after 12 or 24 h of reperfusion in both myocardial I/R injury and hypoxia-reoxygenation (H/R) processes.

While the exact mechanism of NS expression remains elusive, the pathological milieu created by myocardial I/R injury, including local inflammation, hypoxia, and myocardial dysfunction, hints at the potential mechanisms underlying Myocardial I/R injury-induced NS expression. Our RNA-seq results and GSEA analysis indicate enrichment of the GROSS_HYPOXIA_HIF1A_DN pathway and HAN_JNK_SINGALING_DN pathway. Additionally, studies by César Ríos-Navarro et al. [31] demonstrated potent neoangiogenic stimulation by HIF-1α in myocardial infarction, and Shao et al. [6] reported that JNK signalling could reactivate the Akt pathway, thereby alleviating myocardial cell apoptosis. Utilizing the JASPAR tool, we predicted potential binding sites for c-Jun and HIF-1α in the NS promoter region. ChIP-qPCR results revealed significant enrichments of HIF-1α and c-Jun in the promoter regions of NS. Subsequently, qPCR results showed that the inhibition of JNK and HIF-1α resulted in downregulated NS levels at 6 and 12 h of reoxygenation. Immunofluorescence and Western blot analyses further confirmed the downregulation of NS expression following exposure to JNK and HIF-1α inhibitors. These findings strongly suggest that the JNK/c-Jun and HIF-1α pathways induce NS expression in H/R injury. Certain studies have delved into the potential anti-apoptotic mechanism of NS. The seminal work by Tsai et al. [20] uncovered that the interaction between mutant NS and p53 could promote apoptosis in CNS stem cells. Knocking down NS expression was found to elevate p53 and cleaved caspase-3 expression in cardiomyocytes. Our results corroborate these findings by demonstrating that suppressing NS expression exacerbates cell apoptosis, elevated cleaved caspase-3 and Bax expression, and diminishes Bcl2 expression in H/R-induced H9C2 cell injury. Furthermore, GSEA analysis revealed enrichment in the REACTOME_MITOPHAGY pathway. However, the role of autophagy in myocardial I/R injury remains contentious [12, 32]. Our investigations indicated that inhibiting autophagy exacerbates cell apoptosis. Intriguingly, interference with NS expression in H/R-induced injury led to a reduction in the fluorescence density of LC3. Western blot analysis showed a decrease in the transition from LC3I to LC3II, downregulation of Beclin1, and upregulation of p62.

Through integrative analysis of RNA-seq and our functional data, we demonstrated a pivotal role of NS upregulation in the context of myocardial I/R injury. However, it is important to acknowledge that some limitations within our study have affected the strength of our findings. Firstly, only male mice were employed in our I/R model, making it difficult to assess the involvement of sex hormones in NS regulation and cardiac I/R injury. Additionally, the limitation of small sample size and the necessity for optimization of reperfusion periods potentially compromise the reliability and generalizability of our results. Moreover, the mechanisms by which NS exerts its cardioprotective effects remain incompletely explored. Serving as an important stem protein, NS has been reportedly associated with various protective functions, including genetic stability maintenance, telomere repair, p53-dependent anti-apoptotic activity, and tissue regeneration. Further investigations into these aspects may offer valuable insights into the role of NS in cardiac I/R protection.

In summary, this study proposes that the transcription factors c-JUN and HIF-1α can bind to the promoter region of NS, leading to transient upregulation of NS expression. Elevated NS expression initiates autophagy and alleviates apoptosis in cardiomyocytes.

## Materials and methods

### Mice myocardial I/R injury model

One hundred twenty healthy 7 weeks C57BL/6 male mice were procured from the Experimental Animal Center at Nantong University. All procedures were ethically reviewed and approved by the Nantong University Animal Ethics Committee, with the approval number (S20220715-081). The mice were randomly assigned to the following groups (*n* = 3 in each group): the control group received no treatment, while the experimental group underwent left anterior descending coronary artery (LAD) ligation for 30 min followed by reperfusion for 0, 3, 6, 12, and 24 h, respectively (survival rate = 45%). Additionally, all surviving samples were included in the analysis, and mice were randomly assigned to the experiment. However, no blinding was used for group allocation during the experiment.

Briefly, mice were anesthetized by isoflurane using a small animal anesthesia machine (ABS, Yuyan, China) with an oxygen flow rate of induction dose of 1 L/ml, and a maintenance dose of 0.2 L/ml. The mouse was supine and fixed on the surgical board, with shaved hair on the left side of the sternum and iodine wiped. Subcutaneous muscle was bluntly separated, exposing the pleura between the separated third and fourth ribs, followed by tearing open the pleura and the chest cavity with forceps. After the heart was exposed, a loose knot was tied to the middle section of LAD branch of the coronary artery using a 7-0 suture, and then the heart was immediately repositioned before suturing the wound. After 30 min, the knot was untied to initiate reperfusion. Based on the location of the MIRI injury, the left ventricle and apex of myocardial tissue were collected for subsequent experiments.

### Triphenyltetrazolium chloride (TTC) staining and ELISA

After resection, the mouse heart tissues were snap-frozen in a −80 °C refrigerator, followed by cutting them into 2–3 mm thin slices. Subsequently, the slices were stained in a 1% TTC solution in PBS in a dark water bath at 37 °C for 30 min, with gently shaking every 5 min. The infarct size was calculated using Image-Pro Plus. Myocardial infarction indicators, cTn-I, and CK-MB, were assayed using an ELISA kit (MEIMIAN, China).

### Immunohistochemistry

Mouse myocardial tissues were fixed in 4% paraformaldehyde overnight, embedded in paraffin, and cut into approximately 4μm-thick tissue slices. After dewaxing in xylene for 20 min and hydration in various ethanol concentrations for 5 min each, the tissue slices underwent autoclave heating in a citrate repair solution for 20 min to facilitate antigen repair. To block endogenous peroxidase, a 3% hydrogen peroxide solution was applied for 15 min.

The sections were then incubated with a rabbit anti-NS antibody (1:300, ab70346, Abcam) overnight at 4 °C. Subsequently, HPR-conjugated secondary antibody incubation occurred for 2 h at room temperature, followed by incubation with a DAB kit (PWB0167, Proteinbio) for 5 min. After each antibody incubation, the sections were washed with PBS for 5 min (three times). Finally, ethanol was used for dehydration, and the sections were sealed with neutral resin. The sections were examined under an inverted digital microscope (DM5000 B, Leica, Germany).

### Cell culture and RNA interference

H9C2 cells were obtained from the National Collection of Authenticated Cell Cultures of China and were cultured in high-glucose Dulbecco’s modified Eagle’s medium (DMEM, Cytiva, USA) supplemented with 10% fetal bovine serum. The H9C2 cells were recently authenticated by STR profiling and confirmed to be free of mycoplasma contamination. To replicate a sugar-deficient and hypoxic environment, glucose-free DMEM containing 500 μM Cobalt dichloride (CoCl_2_) was employed, and the H9C2 cells were cultured under these conditions for 12 h. Thereafter, the medium was replaced with a standard complete culture medium, initiating the reoxygenation process. All cells were cultured at 37 °C and 5% CO_2_.

For NS interference, H9C2 cells were seeded at 60–70% confluency. 24 h later, the cells were transfected with control siRNA or NS-targeting siRNA (5′-CGU CAC AAC CUC AAA GGU A-3′) using Fugene 6 reagent following the manufacturer’s instrument.

### CCK8

H9C2 cells were plated in 96-well plates at a volume of 100 μl per well and incubated in a glucose-free DMEM medium supplemented with CoCl_2_ for 12 h. CCK8 reagents (abs50003, absin) were then introduced to each well (10 μl per well) and incubated at 37 °C for 2 h. Subsequently, the absorbance was assessed at 450 nm.

### Western blot

Western blot analysis was performed as described previously [33, 34]. The following antibodies were employed: LC3 (14600-1-AP, Protein), NS (ab70346, Abcam), Beclin1 (11306-1-AP, Protein), SQSTM1/p62 (# 23214, Cell Signaling Technology), c-Jun (AF1612, Beyotime), HIF-1α (YT2133, Immunoway), Phospho-c-Jun (AF5779, Beyotime), and GAPDH (60004-1-Ig, Protein).

### Chromatin immunoprecipitation (ChIP) and real-time quantitative PCR (RT-qPCR)

CHIP was performed using the BeyoChIP™ Enzymatic ChIP Assay Kit (P2083S, Beyotime) in accordance with the manufacturer’s protocol. The antibodies used for CHIP assay were as follows: C-Jun P1, 5′-TCCGAGTGGCAGGCATAAAG-3′, and 5′-ACCAAACACCTGACAACATACT-3′; c-Jun P2, 5′-TTCAAAAATTACTTTAGGAGCCAGG-3′, and 5′-AATGCTCCCAACTGGACCAC-3′; c-Jun P3, 5′-CGAATCGAGTGTTTTTAAACTGTGT-3′, and 5′-TTAAGGCACGTTTCTTCGGA-3′; HIFa-1α P1, 5′-TGTAACGTCACGACGAATCGAG-3′, and 5′-CTGTGCCTGGCTCCTAAAGTA-3′; HIF-1α P2, 5′-GAGCAGAAGAGGTCTCAGGT-3′, and 5′TCTTGAGCGCGTGACGTTG-3′; HIF-1α P3, 5′-ACTTTCGAGCCTCTTGCGTT-3′.

For RT-qPCR, total RNA was extracted using the Trizol reagent (Sigma) and then subjected to RT-qPCR experiments using the StepOne Plus Real-Time PCR System (Applied Biosystems, USA). The qPCR reactions were performed using AceQ qPCR SYBR Green Master Mix (Vazyme, China). The primer used for CHIP and RT-qPCR were as follows: NS, 5′-CAGGTTGGAGTGGTTGGTTTC-3′, and 5′-AGCAGGGGAGTTACAAGGTG-3′; β-actin, 5′-AAGTCCCTCACCCTCCCAAAAG-3′, and 5′-AAGCAATGCTGTCACCTTCCC-3′.

### RNA-seq and bioinformatics analysis

Cells underwent 12 h of hypoxia and glucose deficiency, followed by 6 h of reoxygenation. Subsequently, RNA isolation and sequencing were conducted by BGI Genomics, Shenzhen, China. The differential expression profiles (Table S1), relative to the untreated control group, were analyzed using the R package DESeq2. Genes exhibiting |Log2 (Fold-change)| > 0.58 and adjusted *P* value (FDR) < 0.05 were deemed statistically significant. To assess the expression changes in NS and other targets, three public Gene Expression Omnibus (GEO) myocardial I/R animal model datasets (GSE160516, GSE58486, and GSE122020) were utilized. Volcano plots and differential expression profiles were obtained through GEO2R analysis. Furthermore, GSEA_4.1.0 was employed for the Gene Set Enrichment Analysis (GSEA) enrichment analysis.

### Immunofluorescence

H9C2 cells were fixed with 4% paraformaldehyde for 15 min, followed by membrane permeabilization using 0.1% Triton X-100 for 20 min. To block non-specific antigens, cells were treated with 1% BSA for 1 h at room temperature. Subsequently, cells were incubated overnight at 4 °C with NS (1:300, Abcam) and LC3 (1:500, Proteintech). Afterwards, fluorescent secondary antibodies (1:1000, Jackson ImmunoResearch) were applied for 2 h at room temperature, followed by DAPI (P0131, Beyotime) staining for 15 min. Photos were analyzed using a fluorescence microscope (Leica DM5000 B, Germany) and Image J.

### Apoptosis

Cell apoptosis was assessed using an Annexin V-PE/7-AAD apoptosis test kit (AT104, Multi Sciences) following the supplier’s protocol, and flow cytometry (Cytoflex, Beckman) was employed for the detection of cell death.

### Statistic analysis

The results were expressed as mean ± standard deviation (SD) and statistically analyzed using GraphPad Prism 9.0. All data followed a normal distribution as determined by the Shapiro–Wilk test. Groups with similar variances for time- or dose-course effects were analyzed using ordinary one-way ANOVA, while those with unequal variances were analyzed using Welch’s ANOVA. CHIP results were statistically analyzed using an unpaired *t* test. Two-way ANOVA was employed to analyze the statistical differences using two different factors. *P* < 0.05 was considered as statistically significant.

## Supplementary information

Original Western blots

Supplemental materials

Table S1

## Data Availability

The datasets presented in this study can be found in online repositories. The names of the repository/repositories and accession number(s) can be found below: https://www.ncbi.nlm.nih.gov/geo/query/acc.cgi?acc=GSE254950. Other data used and/or analyzed during the current study are available from the corresponding author upon reasonable request.
