# Supplementary material for: Myocardial ischemia-reperfusion injury upregulates nucleostemin expression via HIF-1α and c-Jun pathways and alleviates apoptosis by promoting autophagy
Source: Cell Death Discov. 2024 Oct 30;10:461. doi: 10.1038/s41420-024-02221-x (PMC11525682; doi:10.1038/s41420-024-02221-x)

Figure 1 F

NS

GAPDH

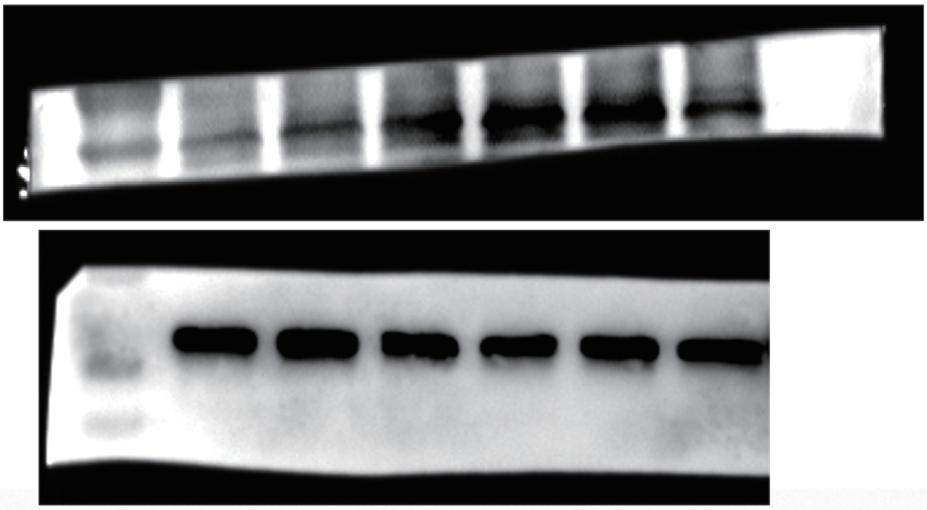

Figure 2 C

NS

GAPDH

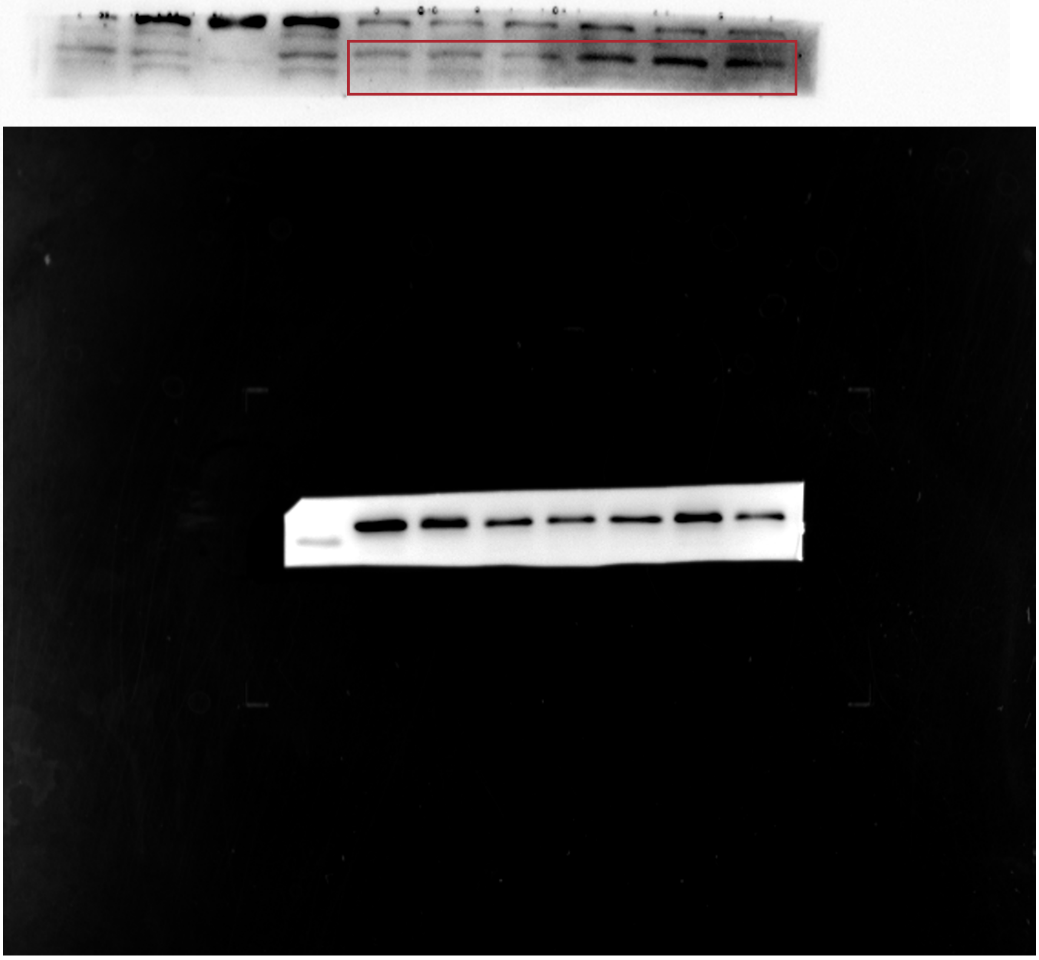

Figure 4 F

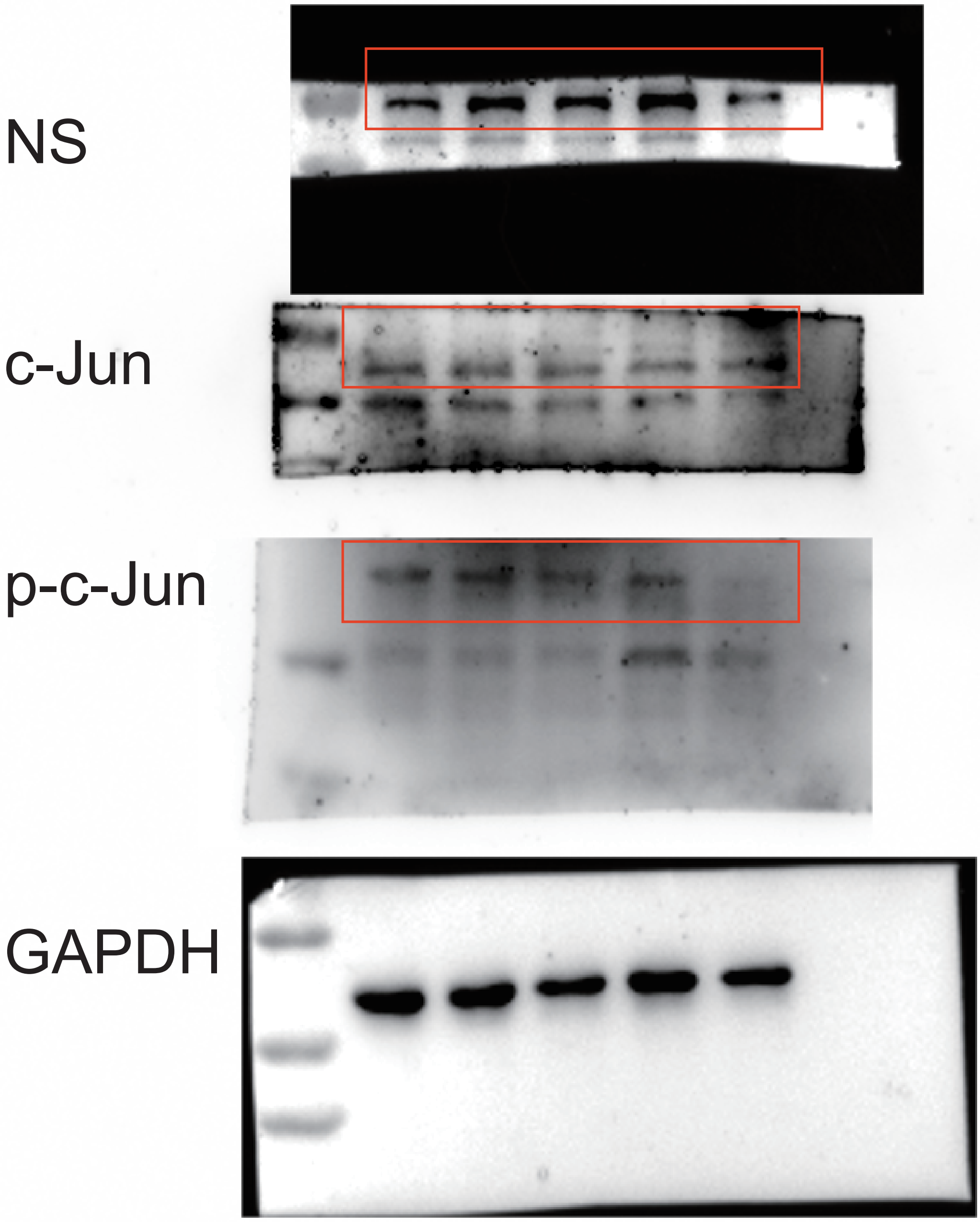

Figure 5-C

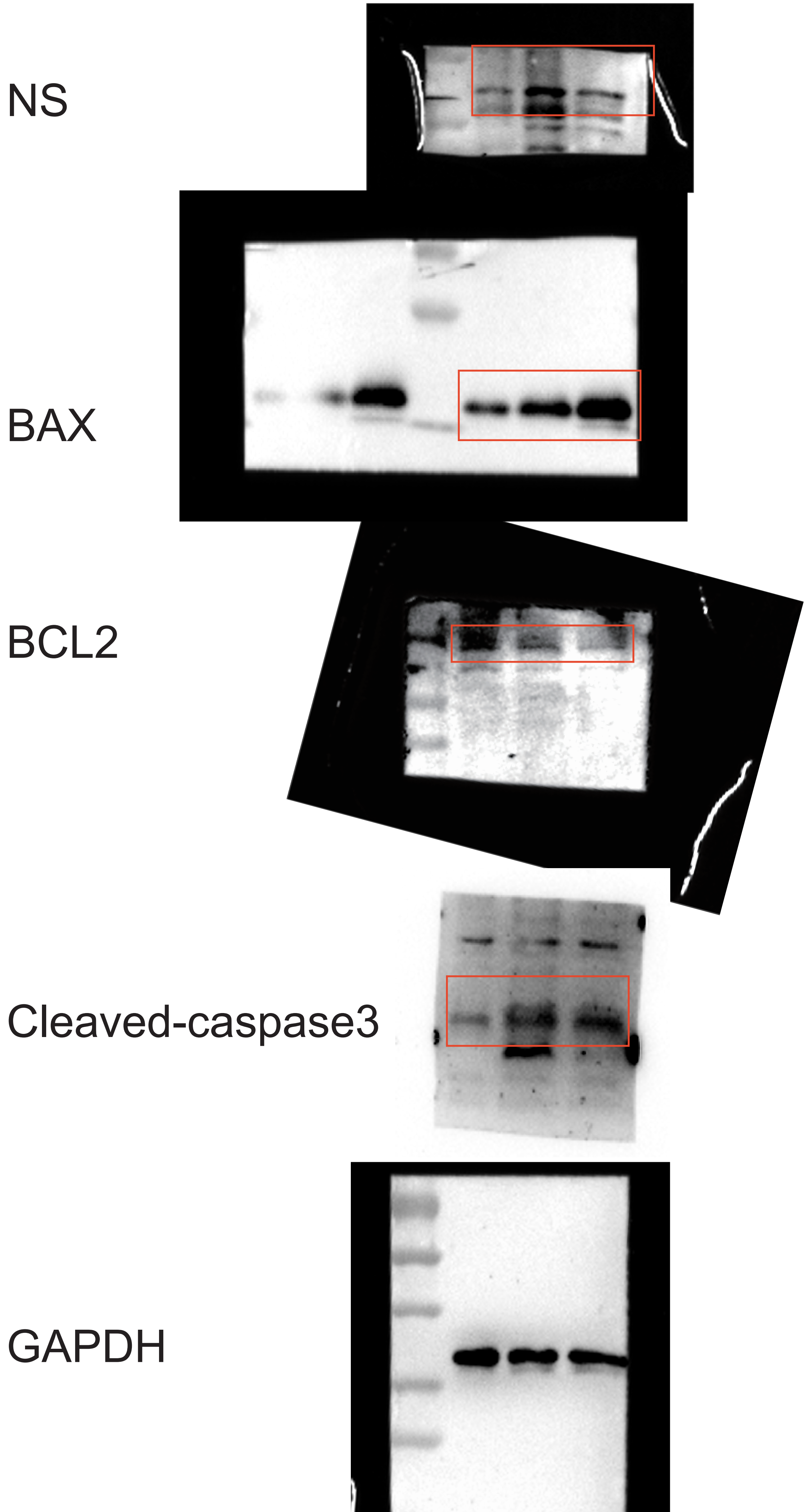

Figure 6-C

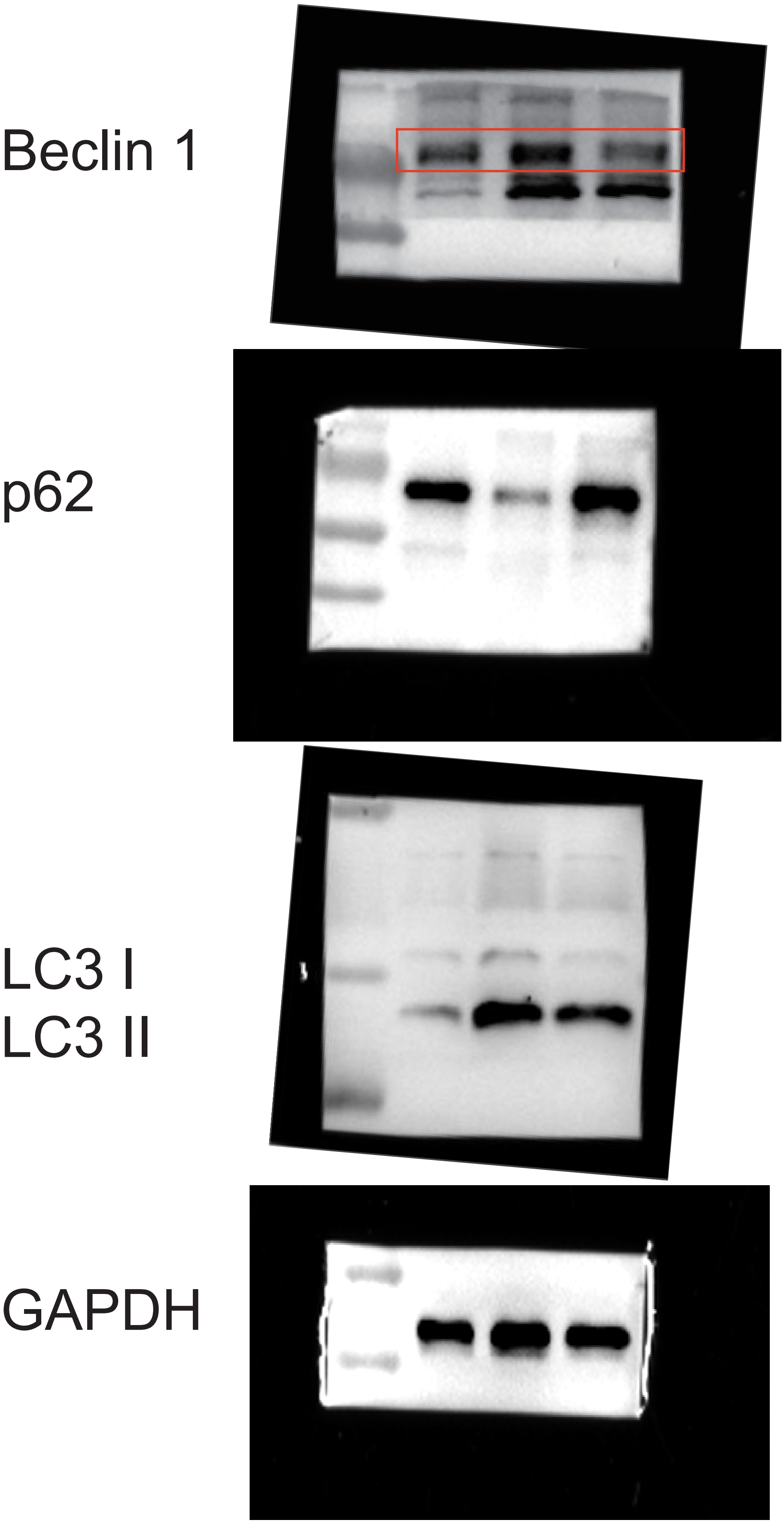

Supplement: Supplementary file 1 — Original Western blots [file 41420_2024_2221_MOESM1_ESM.pdf]
