# Supplementary material for: Myocardial ischemia-reperfusion injury upregulates nucleostemin expression via HIF-1α and c-Jun pathways and alleviates apoptosis by promoting autophagy
Source: Cell Death Discov. 2024 Oct 30;10:461. doi: 10.1038/s41420-024-02221-x (PMC11525682; doi:10.1038/s41420-024-02221-x)
Supplement: Supplementary file 2 — Supplemental materials [file 41420_2024_2221_MOESM2_ESM.pdf]

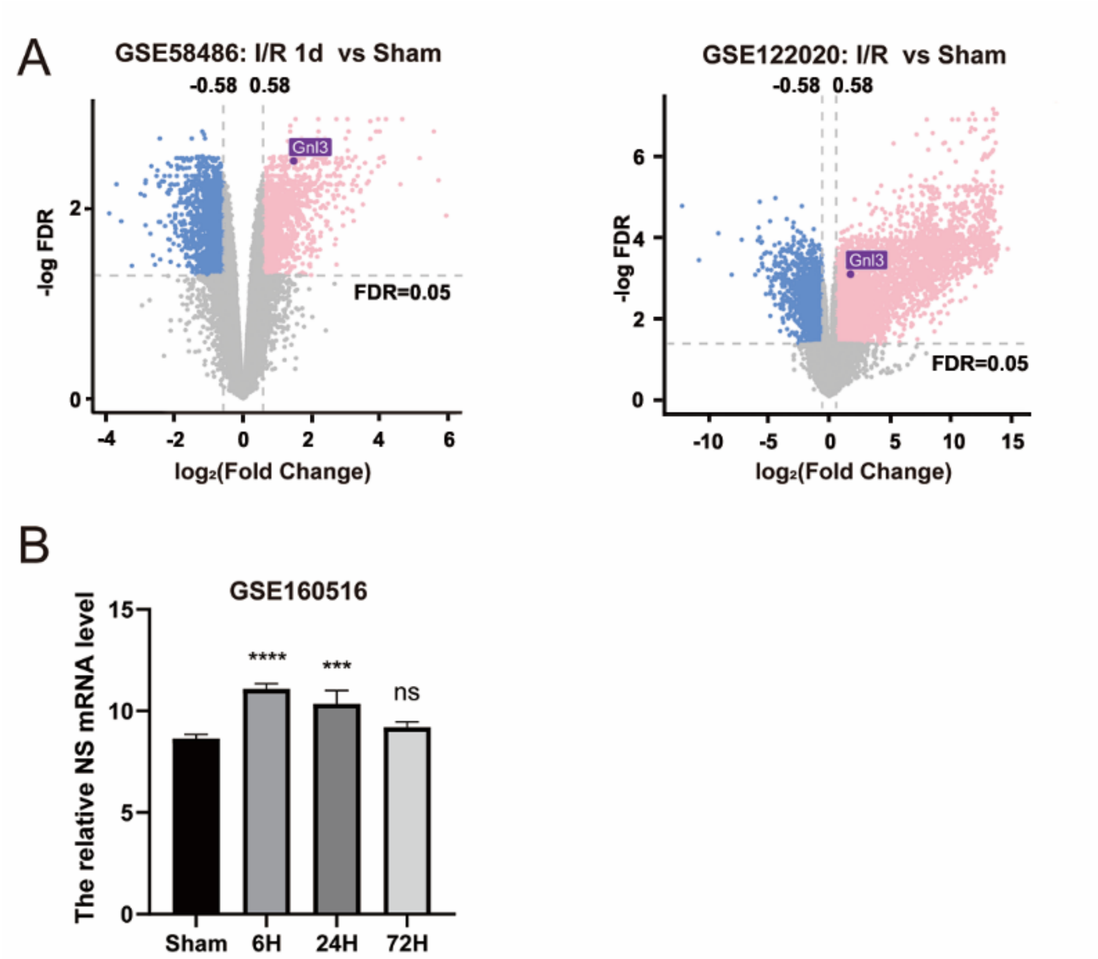

Fig. S1 RNA-seq analysis of three GEO datasets revealed a significant difference between Myocardial I/R injury and the Sham group.

A Volcano plot of differential expression profiles of GEO datasets GSE58486 and GSE122020. **B** Myocardial expression of NS in the Sham group or indicated time points after myocardial I/R injury (GSE160516).
